# Supplementary material for: Auger-spectroscopy in quantum Hall edge channels and the missing energy problem
Source: Nat Commun. 2019 Sep 2;10:3915. doi: 10.1038/s41467-019-11888-1 (PMC6718669; doi:10.1038/s41467-019-11888-1)
Supplement: Supplementary file 1 — Supplementary Information [file 41467_2019_11888_MOESM1_ESM.pdf]

Supplementary Information for "Auger-spectroscopy in quantum Hall edge channels and the missing energy problem"

T. Krähenmann,[1,5,*] S. G. Fischer,[2,3] M. Röösli,[1] T. Ihn,[1] C. Reichl,[1] W. Wegscheider,[1] K. Ensslin,[1] Y. Gefen,[2] and Yigal Meir[3,4]

[1] *Solid State Physics Laboratory, ETH Zürich, CH-8093 Zürich, Switzerland*
[2] *Department of Condensed Matter Physics, Weizmann Institute of Science, Rehovot, 76100 Israel*
[3] *Department of Physics, Ben-Gurion University of the Negev, Beer-Sheva, 84105 Israel*
[4] *The Ilse Katz Institute for Nanoscale Science and Technology, Ben-Gurion University of the Negev, Beer-Sheva, 84105 Israel*
[5] *present address: QuTech and Kavli Institute of Nanoscience, Delft University of Technology, Delft 2628CJ, the Netherlands*

(Dated: August 20, 2019)

Supplementary Note 1: Additional Information on the Transfer Experiment's Energetics

Here we discuss in detail the alignment of the Emitter and Detector QD electrochemical potentials at the specific points indicated by circled numbers in Fig. 3**a** of the main text. That figure is reproduced in Supplementary Figure 1**b**. In addition we show in Supplementary Figure 1**a** the Emitter current measured simultaneously with the Detector current. The current through the Emitter QD is independent of the Detector plunger gate voltage V_{DET}, as expected for the large spatial separation of the Emitter and Detector QDs. Transport through the Emitter QD shows an enhanced current when the Emitter QD electrochemical potential is aligned with the Fermi energy of either the Source or the Reservoir (as indicated by the small black arrows). This enhancement with respect to the current between the two arrows is due to a Fermi edge singularity, studied in detail elsewhere [1]. Except for a slightly larger emission current, the Fermi edge singularity does not qualitatively influence the transfer measurements. The schematic level alignment at the points indicated by the circled numbers in Supplementary Figure 1**b** are shown in Supplementary Figure 1**c**. These numbers used here coincide with the numbers used in the main text.

Supplementary Figure 2 schematically shows the current through the Detector QD as measured in a typical transfer experiment. Characteristic lines and level alignments are indicated to clarify the energetics pertaining to the respective transfer processes (all energies are taken with respect to the grounded Reservoir potential, i.e. $\mu_{\mathrm{Res}} = 0$).

The current through the Sensor QD, shown in Supplementary Figure 3**b** is the same as that shown in Figure 4**a** of the main text. The simultaneously measured current through the Emitter QD is shown in Supplementary Figure 3**a**. Due to the increased bias on the Source contact for this measurement, ($V_{\mathrm{Source}} = -700\ \mu$V) as well as the separation between Sensor and Emitter QDs, which is smaller than that between Emitter and Detector QD, we see a slight tilt in the horizontal lines indicating the onset of current. The enhanced current for $V_{\mathrm{EM}} \approx -0.705$ V stems from an excited state in the Emitter QD and does not show a qualitative influence on the effects described in the main text. The influence of excited states, especially in the Detector QD will be discussed in the next section. The schematic level alignment of those points indicated by the circled numbers in Supplementary Figure 3**b** are shown in Supplementary Figure 3**c**. The numbers used here coincide with the numbers used in the main text.

Supplementary Note 2: Filling Factor Dependence of the Auger Recombination

Supplementary Figure 4 shows characteristic transfer measurements for filling factors (**a**) ν=2 and (**b**) ν=4. To keep the tunnel coupling of the QDs to the leads at the same order of magnitude the voltages applied to the surface gate electrodes have to be adapted accordingly. A quantitative comparison between the measurements is thus not feasible. However, one can detect both the direct transfer features, as well as the Auger-like recombination at varying filling factors.

Supplementary Note 3: Contribution of Excited States to the Detected Transfer

Here we show further experimental results which illustrate the influence of excited states in transfer experiments. Supplementary Figure 5**a** shows a Coulomb-blockade diamond measurement of the Detector QD in a gate-voltage configuration (different from the configurations in the experiments appearing in the main text) showing many excited states. Panel **b** shows the line-cut indicated in panel **a**. Clear steps

* corresponding author: tobiaskr@phys.ethz.ch

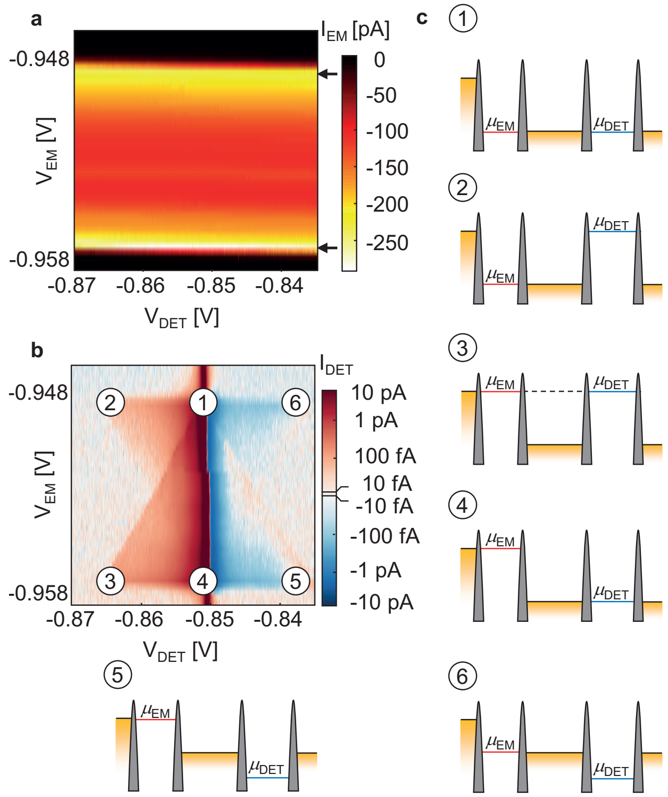

Supplementary Figure 1. **Spectroscopy of electron transfer with quantum Hall edge channels.** **a** 2D colour plot of the current through the Emitter QD for varying Detector (x-axis) and Emitter plunger gate voltage (y-axis). **b** Current through the Detector QD measured simultaneously to **a**. Panel **b** coincides with Fig. 3**a** of the main text. **c** Schematic level alignment of the Emitter and Detector energies indicated in **b**.

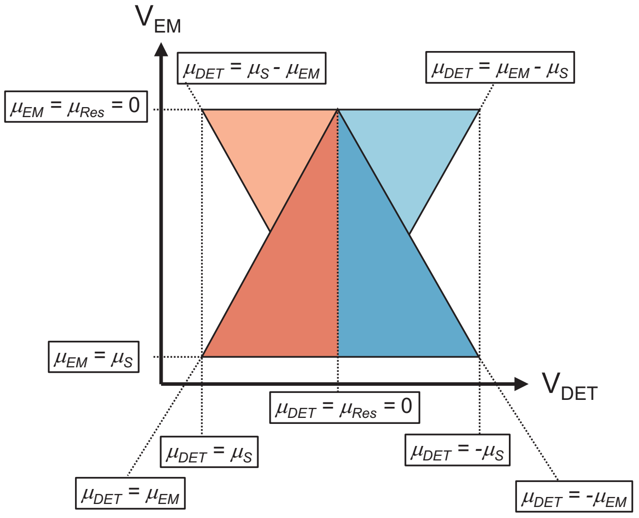

Supplementary Figure 2. **Schematics of the transferred current.** Schematic illustration of the current measured through the Detector QD. Lines of high symmetry and characteristic energetic alignments are highlighted and labelled. All energies are taken with respect to the grounded Reservoir potential, i.e. $\mu_{\rm Res} = 0$.

in the current are observable, which are characteristic for excited states in transport. The vertical lines on the top of the figure indicate the positions of the excited states and will be used for future reference. In panel **c-f** the current through the Detector QD is shown for increasing Source bias from -400 to -700 μV (as indicated on the top of each panel). The voltages applied to the Emitter and Detector plunger gate are scaled accordingly to capture the full transfer measurement. For each panel the comb of vertical lines is scaled to match the plunger gate voltage axis, and shifted such that the solid line matches the gate voltage where the ground state is in resonance with the Fermi energy of the Reservoir (such that e.g. the solid line is on top of line ①-④). The dashed lines thus coincide with the gate voltages at which the respective excited states are in resonance with the Fermi energy of the grounded Reservoir contact. Increasing the bias voltage of the Source contact (i.e. from panel **c-f**) changes the energies involved in the transfer process and gives access to higher lying states. On top of the standard transfer features which we usually observe in the Detector QD current, we can clearly resolve a prominent positive (red) current in the region where we would expect to measure a negative signal. The gate voltages at which these positive features appear clearly correlate with the energies of the excited state in the Detector QD as seen when increasing the Source bias. To understand why an excited state can lead to a positive current signal, we have to look at the level alignment of the Detector QD, which is shown in the inset of Supplementary Figure 5**b**. The depicted situation corresponds to a plunger gate voltage of roughly $V_{\rm DET} \approx -0.457$ V (in all four panels). The electrochemical potentials of the ground state as well as the first excited state are below the Fermi energy of the Reservoir contact. Thus, in equilibrium the ground state should be occupied by an electron. As long as the ground state stays occupied, the excited state transitions are energetically forbidden due to Coulomb repulsion. We have seen that the electron of the ground state can tunnel into an empty state of the Reservoir, which was generated by electron-electron collisions. As soon as the ground state is emptied, all the excited state transitions suddenly are allowed, and conventional transfer of electrons (from the Reservoir to the Drain) through an excited state becomes possible as long as the ground state stays empty. If the ground state becomes populated all excited state transitions are again energetically forbidden. This phenomenon resembles the Pauli-Spin blockade where transport switches between a blocked and unblocked situation due to different spin states [2]. Here the switching happens between the two transport directions Drain to Reservoir (through the

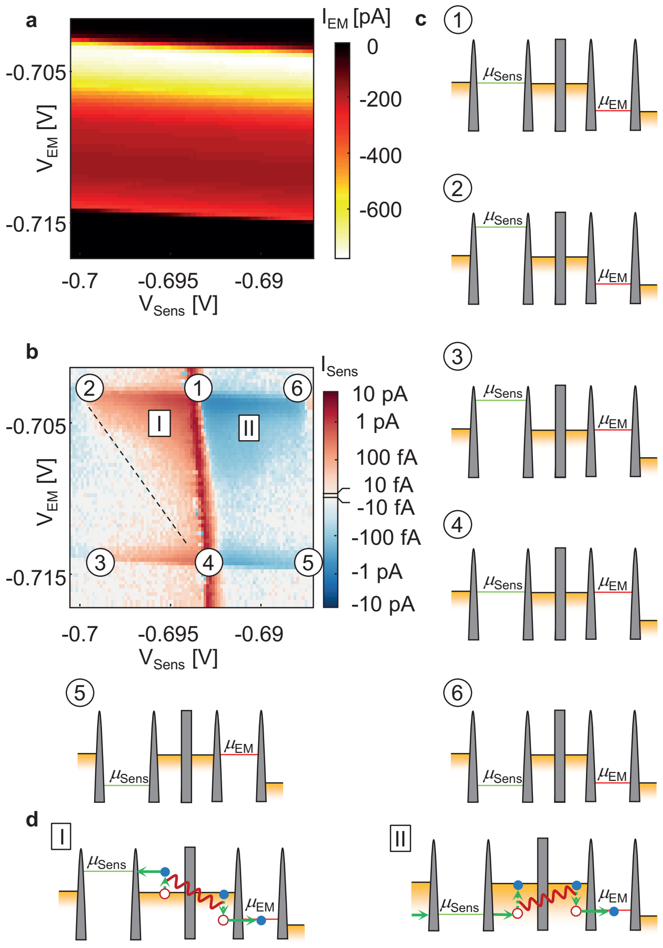

Supplementary Figure 3. **Spectroscopy of Auger-like processes employing a Sensor QD.** **a** 2D colour plot of the current through the Emitter QD for varying Sensor (x-axis) and Emitter plunger gate voltage (y-axis). **b** Current through the Sensor measured simultaneously to **a**. (This panel is the same as in the main text Figure 4**a**.) **c** Schematic level alignment of the situations labelled in **b**. **d** Schematic description of the Auger-like scattering processes detected in the experiment.

ground state) and vice versa (over an excited state). The measured sign of the net current depends on both the tunnel coupling to the different orbital states, as well as the amount of electrons which pass through the Emitter QD.

Supplementary Note 4: Electrical Isolation of the Sensor QD

To confirm that there is no charge transfer between the Source and *Right* contacts of the sample, we show the sum of the currents measured in terminals *Left* (corresponding to I_{Sens}) and *Right* in Supplementary Figure 6. Electrons tunneling from the Source to the *Right* side of the heterostructure will have to relax in

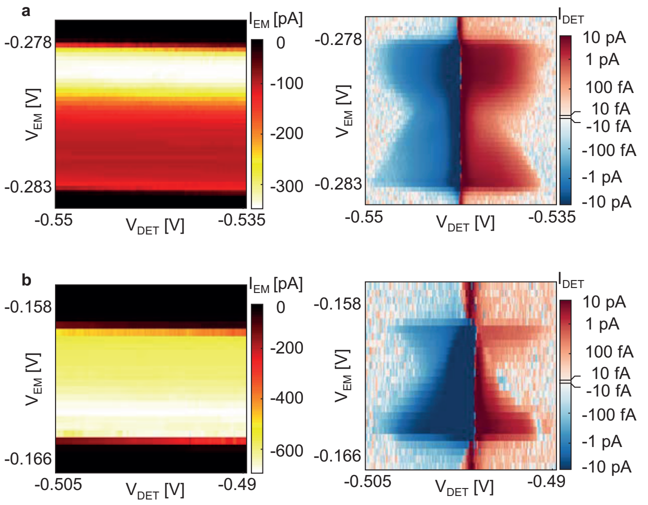

Supplementary Figure 4. **Transfer Measurements for different Filling Factors.** 2D colour plots of the current through the Emitter QD (left) and Detector QD (right) for varying Detector (x-axis) and Emitter plunger gate voltage (y-axis) at a bulk filling factor **a** ν=2 and **b** ν=4.

either the *Left* or the *Right* contact and thus would be visible in Supplementary Figure 6 as a positive current. Two features are visible in the combined current, both of which do not show charge transfer from Source to *Right*. At the vertical line at $V_{Sens} \approx -0.693$ V the Sensor QD electrochemical potential is in resonance with the electrochemical potential of the *Left* and *Right* contact. The vertical signal observed there is similar to the background contribution shown in the main text Figure 2. The second feature is a horizontal region of negative current (around V_{EM} = -0.705 V) and corresponds to a concomitant high current through the Emitter QD (see Supplementary Figure 3a) and is due to a capacitive coupling of the output of the corresponding IV-converters used in the experiment.

Supplementary Note 5: Additional Information on the Parameters in the Theoretical Model

The main purpose of the theoretical model, treating Source, Reservoir, Drain, *Right* and *Left* regions as single and parallel channels, is to demonstrate that interactions between electrons in these different regions of the sample generate current in triangles ①-②-④ and ①-④-⑥ in Fig. 3b of the main text, and in all of the triangles in Fig. 4b of the main text. Thereby, our theoretical analysis supports the conclusion that these currents are a result of Auger-like recombination processes. Interactions between electrons on the same edge in the reservoir region qualitatively account for the current in ①-③-④ and ①-④-⑤. The dominant relaxation mechanism for currents in the latter two triangles is likely due to interactions between electrons on

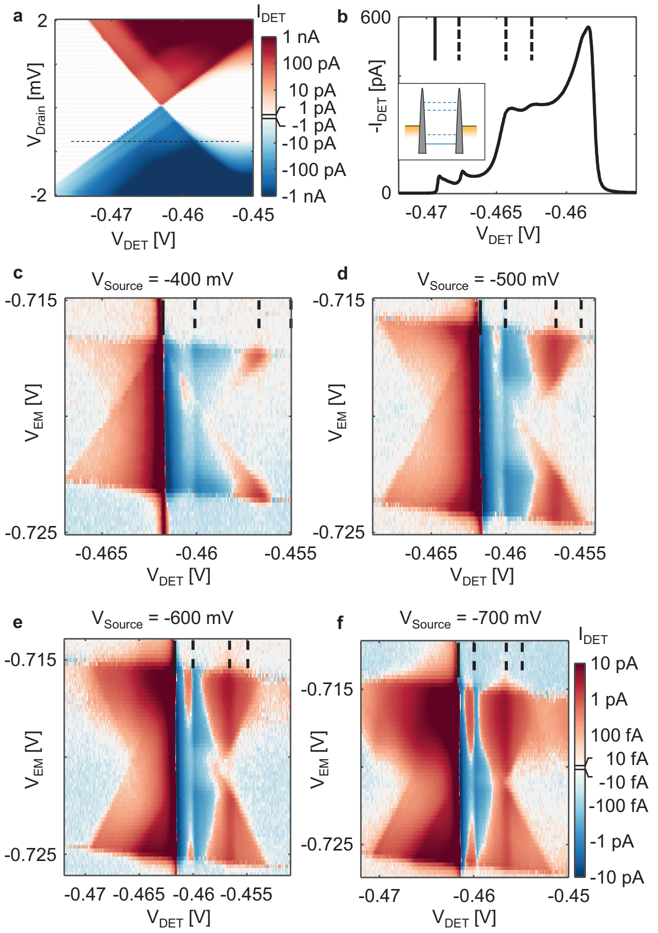

Supplementary Figure 5. **Transfer through excited states.** **a** Coulomb-blockade diamond measurement of the Detector QD. **b** Detector current at $V_{\mathrm{Drain}} = -800\ \mu$V (dashed line in **a**). The vertical lines on the upper part of the panels indicate the spacing between the ground state (solid line) and the first three excited states (dashed lines). Inset: level schematics of the Detector QD with excited states. **c-f** Current through the Detector QD measured in a transfer experiment for different bias voltages on the Source contact (indicated in the upper part of each panel). Upon increasing the bias voltage, the plunger gate voltage ranges have to be extended. The vertical lines on the top side of each panel are scaled accordingly and show the respective energies of the excited states (i.e. the dashed line indicates the Detector plunger gate voltage at which the excited state crosses the Fermi energy of the Reservoir). The colour scale indicated in panel**f** applies also to panels **c**-**f**.

different channels on the same edge, which goes beyond the scope of our treatment and has been considered in detail in Refs. [3–8]. Such interactions alone cannot account for inter-edge Auger recombination.

The model interaction matrix element between electrons at a distance $|r|$ in the same channel is given by $U(|r|) = \nu \exp(-|r|/\lambda)/2\lambda$. The prefactor of this interaction is chosen such that in the limit $\lambda \to 0$ a contact interaction with strength ν is obtained, as is

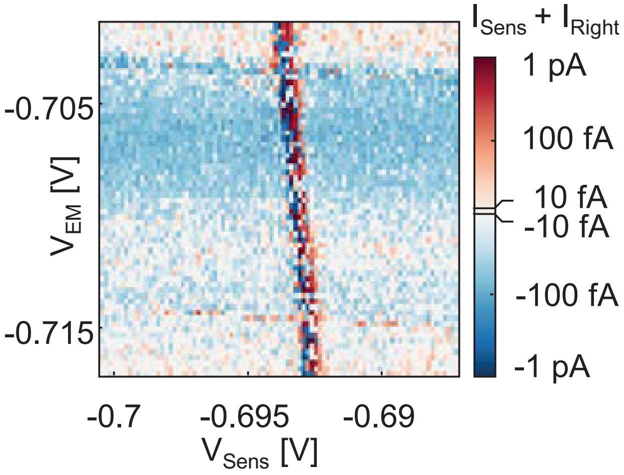

Supplementary Figure 6. **Electrical isolation of the Sensor QD** 2D colour plot of the sum of the currents measured in terminals *Left*and *Right*.

regularly employed in theoretical studies of relaxation in quantum Hall edges [5–8]. Furthermore, the Fourier transform of the interaction U is compatible with the approximation for small photon momenta that is considered, e.g., in Refs. [3, 6]. The specific form of the screened interaction does not have a qualitative impact on the calculated current. To take into account phenomenologically the additional separation between electrons in different channels, the interaction U is further suppressed by a factor $\exp(-d_i/\lambda)$, where d_i indicates the distance between the respective channels (estimated from the point of injection at the Emitter QD).

Parameter values for tunneling rates, bias, Fermi velocity and distances, that generate the plots in Figs. 3b and 4b of the main text, are oriented to values encountered in typical samples, and listed in Supplementary Table 1. All features in these Figures are stable with respect to variations of all listed parameters. The parameters for interaction strength ν and screening length λ, that enter the above-described screened electron-electron interaction, are not easily accessible in the experiment. The combination for ν and λ employed in Fig. 3b and Fig. 4b of the main text generates a comparable ratio of currents due to inelastic processes to Emitter current $e\Gamma/2\hbar$ as observed in the experiment, and assumes a screening length that is short in comparison to sample dimensions.

tunneling rate through QDs	Γ	1μeV
source - drain bias	$\Delta\mu$	400μeV
Fermi velocity	v	$\sim 10^{-5}$ m/s
Coulomb interaction strength	ν	$\sim 2.7 \cdot 10^{-5}$m/s
separation between QDs	x	2μm
screening length	λ	$.25\mu$m
reservoir - source distance	d_1	$.7\mu$m
Right - source distance	d_2	$.7\mu$m
Right - reservoir distance	d_3	1μm

Supplementary Table 1. Parameters employed to generate Figs. 3**b** and 4**b** of the main text.

[1] T. Krähenmann, L. Ciorciaro, C. Reichl, W. Wegscheider, L. Glazman, Thomas Ihn, and K. Ensslin, New Journal of Physics **19**, 023009 (2017).

[2] K. Ono, D. G. Austing, Y. Tokura, and S. Tarucha, Science **297**, 1313 (2002).

[3] J. T. Chalker, Y. Gefen, and M. Y. Veillette, Physical Review B **76**, 085320 (2007).

[4] I. P. Levkivskyi and E. V. Sukhorukov, Physical Review Letters **103**, 036801 (2009).

[5] A. M. Lunde, S. E. Nigg, and M. Büttiker, Physical Review B **81**, 041311 (2010).

[6] D. L. Kovrizhin and J. T. Chalker, Physical Review B **84**, 085105 (2011).

[7] D. L. Kovrizhin and J. T. Chalker, Physical Review Letters **109**, 106403 (2012).

[8] D. Ferraro, B. Roussel, C. Cabart, E. Thibierge, G. Fève, C. Grenier, and P. Degiovanni, Physical Review Letters **113**, 166403 (2014).
